# Single‐cell transcriptomic analysis of the immune response to CHIKV infection

**DOI:** 10.3389/fimmu.2026.1979359

**Published:** 2026-10-29

**Authors:** Peng Huang, Huiyi Wang, Siya Xu, Miaogen Li, Mingjuan Guo, Haiyan Wang, Xiaoyan Gou, Can Wang, Yanjun He, Wei Pan

**Affiliations:** 1 Women and Children Medical Research Center, Affiliated Foshan Women and Children Hospital, Guangdong Medical University, Foshan, China; 2 School of Pharmaceutical Sciences, Southern Medical University, Southern Medical University, Guangzhou, China; 3 Biobank, Affiliated Foshan Women and Children Hospital, Guangdong Medical University, Foshan, China; 4 Foshan Key Laboratory of Stem Cell and Maternal-Child Health, Foshan Women and Children Hospital, Foshan, China; 5 Internal Medicine Department, Affiliated Foshan Women and Children Hospital, Guangdong Medical University, Foshan, China; 6 Shenzhen Digital Cloud Valley Biotechnology Co., Ltd, Shenzhen, China; 7 Emergency Department, Affiliated Foshan Women and Children Hospital, Guangdong Medical University, Foshan, China; 8 Geriatric Department, Affiliated Foshan Women and Children Hospital, Guangdong Medical University, Foshan, Guangdong, China

**Keywords:** antiviral, Chikungunya virus (CHIKV), immune response, interferon response, single-cell RNA sequencing (scRNA-seq)

## Abstract

**Background:**

The Chikungunya virus (CHIKV), a re-emerging mosquito-borne alphavirus, is responsible for acute febrile illness and severe polyarthralgia. Although both innate and adaptive immune responses influence the disease outcomes, the detailed cellular immunopathogenesis of CHIKV in peripheral blood is not yet fully elucidated.

**Methods:**

We profiled PBMCs from 29 patients with acute CHIKV infection and 10 controls via single-cell RNA sequencing. Between-group analyses treated donors as biological replicates, incorporating pseudobulk differential expression analysis as well as donor-level comparisons of pathway scores and cell proportions. Orthogonal validation was performed using qPCR and ELISA, and an exploratory analysis was conducted on a publicly available DENV dataset.

**Results:**

Interferon-response signatures were upregulated across the majority of PBMC populations, with particularly prominent signals observed in monocytes and NK cells. Differences in relative cell composition were detected in descriptive statistics, but did not retain statistical significance after donor-level multiple testing correction. B-cell immunoglobulin isotypes varied across cell subsets, and their transcripts did not allow determination of antigen specificity. Cross-study analysis of DENV did not support the quantitative conclusion that the CHIKV-induced immune response is stronger.

**Conclusions:**

Acute CHIKV infection is associated with a broad interferon-stimulated transcriptional program in PBMCs. Conclusions drawn from cross-sectional and cross-study comparisons require cautious interpretation.

## 1 Introduction

The Chikungunya virus (CHIKV), a re-emerging mosquito-borne alphavirus, poses a significant and increasing threat to global public health, having caused millions of infections worldwide (1–3). The clinical presentation of the infection typically involves an acute febrile illness, often accompanied by rash, severe polyarthralgia, and myalgia (4). Notably, approximately 50% of affected individuals develop chronic arthritis, which can persist for several months (5).

Host immune responses are critical in determining the outcome of CHIKV infection (6). An immediate innate immune response, particularly the type I interferon (IFN) response, is essential for controlling acute viral replication (7). However, CHIKV has evolved mechanisms to evade host immune defenses; notably, the viral nonstructural protein 2 (nsP2) disrupts STAT1 signaling and inhibits host transcriptional processes (8, 9). When viral replication exceeds immune control, excessive inflammation may occur, potentially contributing to severe disease manifestations. Unregulated inflammation has been linked to adverse outcomes in CHIKV infection (6). These findings highlight the importance of elucidating the mechanisms by which host immunity detects and responds to CHIKV infection.

Recent studies emphasize the need for detailed immune profiling in viral infections. In COVID-19, severe cases are linked to myeloid cell issues (10), while dengue virus (DENV) has distinct immune markers for severe disease (11). These findings highlight the importance of single-cell analysis in understanding viral immunopathology. Previous work has characterized CHIKV immunity using cytokine profiling, bulk transcriptomics, and cellular immune phenotyping. More recently, single-cell transcriptomic studies in non-human primate models have resolved acute splenic and peripheral immune responses as well as hepatic immune alterations during CHIKV infection (12, 13). Human single-cell datasets, particularly from larger acute cohorts, remain comparatively limited. We therefore performed scRNA-seq of PBMCs from 28 patients with acute CHIKV infection and 10 healthy controls to define the circulating immune landscape at cellular resolution.

In this study, we profiled PBMCs from patients with acute CHIKV infection to characterize circulating immune responses at single-cell resolution. We assessed transcriptional and compositional changes at the donor level, and utilized a publicly available DENV cohort exclusively to explore shared and dataset-specific patterns. Since the included cohorts were matched neither clinically nor technically, the cross-study analysis was not applied to rank the magnitude of immune response induced by different viruses.

## 2 Results

### 2.1 Overall analysis of single-cell PBMC transcriptomic profiling for CHIKV infected cases

To elucidate the immunological landscape of PBMCs in individuals infected with CHIKV, we conducted scRNA-seq utilizing the 10x Genomics platform on PBMC samples obtained from 10 healthy donors and 29 CHIKV-infected patients (**Figure 1A**). Rigorous quality control measures were applied to the single-cell dataset to exclude low-quality cells and genes with low expression levels, resulting in the retention of 414,257 cells and 35,168 genes for subsequent analyses. Through unsupervised clustering, we delineated 10 principal immune and hematopoietic cell populations, characterized by the expression of canonical marker genes. These populations comprised CD4^+^ T cells, CD8^+^ T cells, B cells, plasma cells, natural killer (NK) cells, γδ T cells, dendritic cells (DCs), monocytes, erythroid cells, and platelets (**Figures 1B, C**). The distribution of cells across samples was relatively balanced, thereby minimizing potential biases in downstream analyses (**Figure 1D, G**).

**Figure 1:**
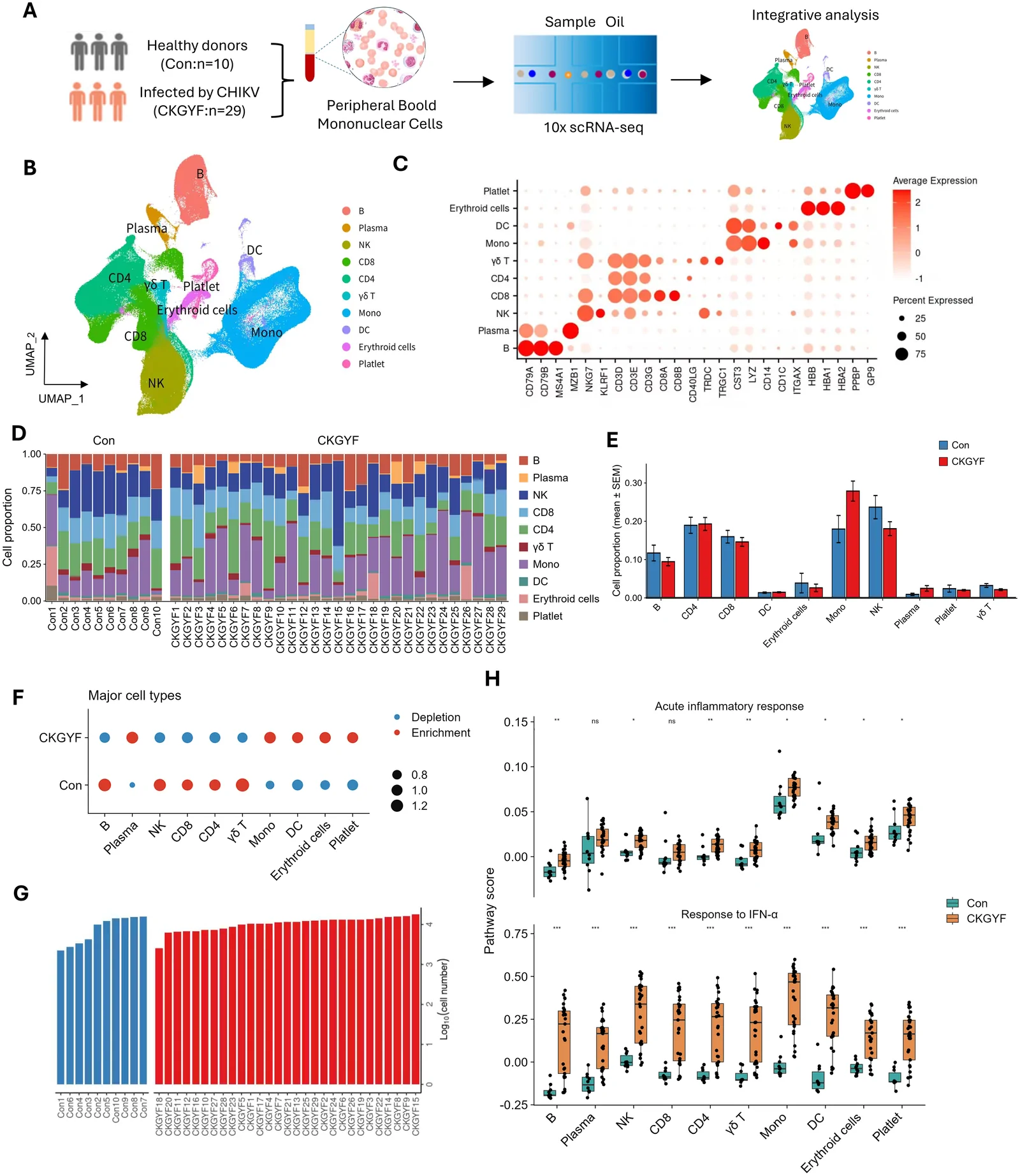
Single-cell atlas of circulating immune and haematopoietic cells in acute CHIKV infection. **(A)** Study design and single-cell RNA-sequencing workflow for peripheral blood mononuclear cells (PBMCs) from 10 healthy controls (Con) and 29 patients with acute chikungunya virus infection (CKGYF). **(B)** Uniform manifold approximation and projection (UMAP) of the PBMC atlas, coloured by the ten annotated populations. **(C)** Canonical marker expression across populations; dot size indicates the percentage of expressing cells and colour indicates scaled average expression. **(D)** Relative cell-type composition of each donor. **(E)** Mean donor-level cell-type proportions; error bars indicate s.e.m., as labelled. **(F)** Pooled observed-to-expected cell-type ratios in each group. Dot size represents the ratio; red and blue indicate enrichment and depletion relative to expectation, respectively. **(G)** Number of cells contributed by each donor, displayed on a log10 scale. **(H)** Donor-level acute inflammatory-response and response-to-interferon-alpha module scores across populations. Group comparisons used two-sided Wilcoxon rank-sum tests with Benjamini-Hochberg correction within each panel, as specified in Methods. ns, not significant; *P ≤ 0.05, **P ≤ 0.01, ***P ≤ 0.001. NK, natural killer; DC, dendritic cell; Mono, monocyte; gamma-delta T, gamma-delta T cell.

Pooled observed-to-expected ratios suggested differences in relative PBMC composition (**Figure 1F**). We therefore compared the proportion of each cell type across 29 patients and 10 control donors (**Figure 1E**). None of the ten major cell populations remained significant after Benjamini–Hochberg correction (all adjusted *P* ≥ 0.234), so the pooled ratios are presented only for descriptive purposes. In contrast, donor-level response-to-IFN-α scores were higher in the CHIKV group across all major cell types (**Figure 1H**). Acute inflammatory-response scores varied among different cell types, and did not remain significant for every population after correction. It should be noted that these proportions do not reflect absolute blood cell counts.

### 2.2 Strong interferon responses were observed in innate immune cells

To elucidate the regulatory effects of CHIKV infection on host innate immune cells, monocytes and DCs were further stratified into distinct subpopulations. Our analysis identified multiple transcriptionally distinct subsets of innate immune cells, including NK cells, CD1C^+^ conventional dendritic cells (cDC2), plasmacytoid dendritic cells (pDCs), CD14^+^ classical monocytes (Mono_CD14), CD16^+^ non-classical monocytes (Mono_CD16), and NEAT1-high monocytes (Mono_NEAT1) (**Figures 2A, B**). We evaluated the subset-specific enrichment preferences of innate immune cells in response to CHIKV infection. Observed-to-expected ratios indicated changes in innate immune cell composition, but donor-level proportions failed to show consistently significant differences after correction (**Figures 2C, D**). Donor-aggregated inflammatory scores were higher in the CHIKV group across all tested innate immune subsets (**Figure 2E**).

**Figure 2:**
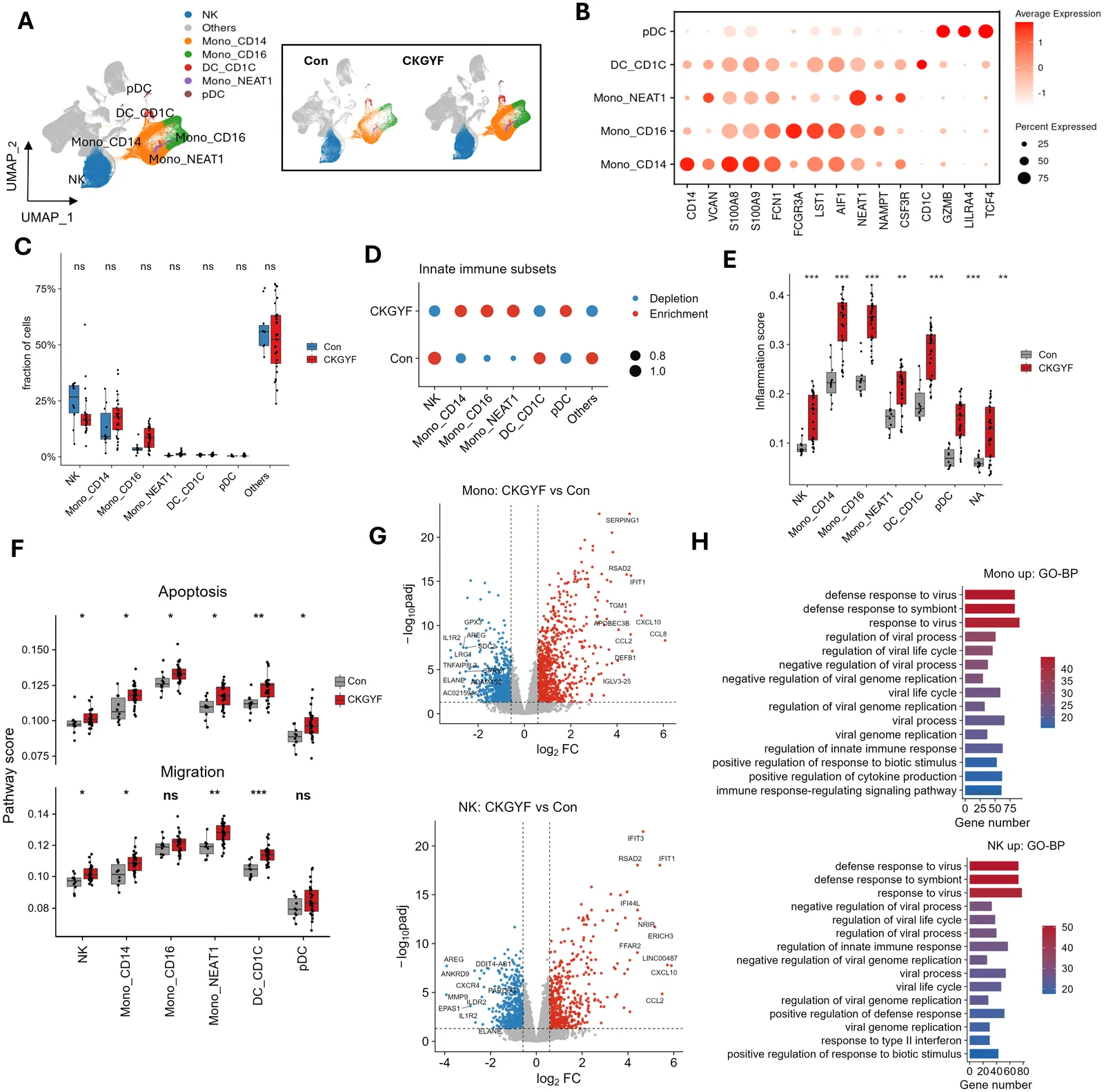
Innate immune-cell composition and transcriptional signatures in acute CHIKV infection. **(A)** UMAP highlighting NK cells, CD14-positive monocytes (Mono_CD14), CD16-positive monocytes (Mono_CD16), NEAT1-high monocytes (Mono_NEAT1), CD1C-positive dendritic cells (DC_CD1C) and plasmacytoid dendritic cells (pDCs), with embeddings separated by condition. Other populations are grey. **(B)** Marker expression across myeloid subsets; dot size indicates the percentage of expressing cells and colour indicates scaled average expression. **(C)** Donor-level proportions of the indicated populations. **(D)** Pooled observed-to-expected ratios; dot size denotes the ratio, with red indicating enrichment and blue depletion. **(E)** Donor-level inflammatory-response scores. **(F)** Apoptosis and migration module scores, averaged within each donor and population. **(G)** Differential expression in monocytes and NK cells, comparing CHIKV with controls. Red and blue denote increased and decreased expression, respectively; selected genes are labelled. **(H)** Gene Ontology biological-process enrichment among genes increased in monocytes and NK cells; bar length denotes gene count. The study cohort comprised 10 controls and 29 patients; evaluable donor numbers depend on population recovery.

When analyzing monocytes and NK cells, donor-level pseudobulk differential expression analysis detected the enrichment of interferon-stimulated genes and antiviral pathways (**Figures 2G, H**). Module scores associated with cell migration and apoptosis are presented as transcriptional signatures, with donor-level effect estimates shown in revised **Figure 2F**.

### 2.3 Immunological features of B cell subsets

To elucidate dynamic changes among different B cell subtypes, we identified six transcriptionally distinct B cell subsets: naive B cells (B_Naive), germinal center B cells (B_GC), memory B cells (B_Memory), intermediate memory B cells (B_iMemory), plasmablasts (B_Plasmablast), and plasma cells (B_Plasma) (**Figures 3A, B**). These six B-lineage subsets were successfully separated. After correction, donor-level comparisons of their relative proportions did not retain statistical significance, and the cross-sectional distribution data cannot confirm the existence of a differentiation trajectory from naive B cells to plasmablasts (**Figure 3C**). Pseudobulk analysis confirmed interferon-stimulated transcriptional changes in the B/plasma cell compartment (**Figures 3D, E**).

**Figure 3:**
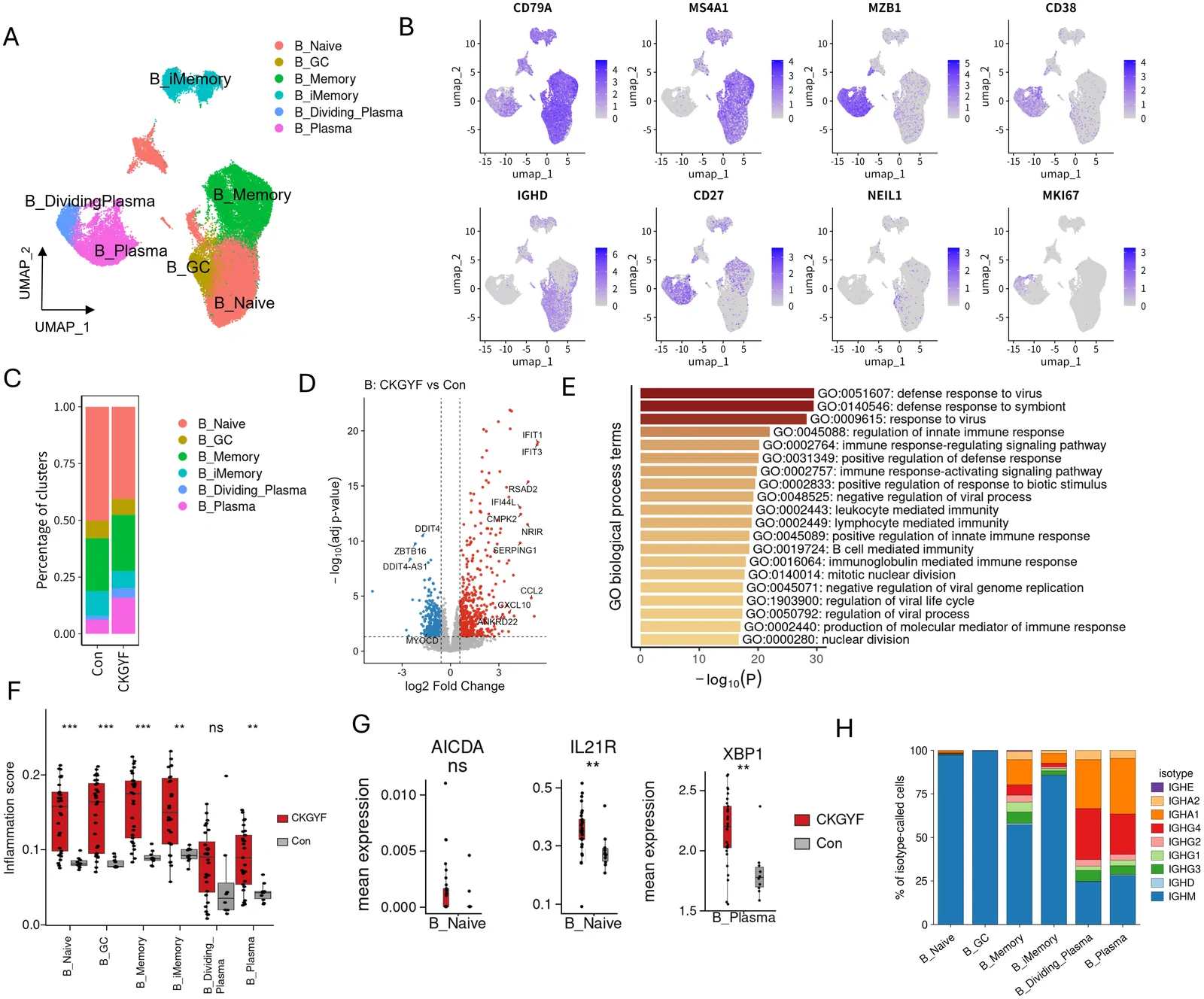
B-lineage transcriptional states and immunoglobulin heavy-chain profiles in acute CHIKV infection. **(A)** UMAP of B-lineage cells, coloured by the six displayed cluster labels: B_Naive, B_GC, B_Memory, B_iMemory, B_Dividing Plasma and B_Plasma. **(B)** Expression of CD79A, MS4A1, MZB1, CD38, IGHD, CD27, NEIL1 and MKI67 on the same embedding. **(C)** Pooled proportions of the six clusters within each condition. **(D)** Differential expression between CHIKV and controls in the B-lineage compartment; red and blue indicate increased and decreased expression, respectively. **(E)** Gene Ontology biological-process enrichment, with bar lengths showing -log10(P). **(F)** Donor-level inflammatory-response scores across B-lineage subsets. **(G)** Donor-level mean expression of AICDA and IL21R in naive B cells and XBP1 in plasma cells. **(H)** Distribution of immunoglobulin heavy-chain isotypes among isotype-called cells in each subset. Methods specify assignment to the most abundant heavy-chain constant-region transcript in cells with at least ten total heavy-chain unique molecular identifiers. Isotype composition does not establish antigen specificity. The study cohort comprised 10 controls and 29 patients.

Immunoglobulin heavy-chain profiles varied across B cell subsets and plasma cells, but transcript isotype information cannot be used to determine antigen specificity (**Figure 3H**). The donor-level comparison of *AICDA* expression in naive B cells was not significant after correction (**Figure 3G**). The signals of *S100A8*, *S100A9* and *S100A1*2 detected in B cells were consistent with residual ambient RNA, so these genes were not used as evidence for intrinsic inflammatory activation in B cells. The revised B-cell inflammation score excludes the ten genes susceptible to ambient RNA contamination (**Figure 3F**).

### 2.4 Features of T cell subsets in patients with CHIKV

After per-sample doublet filtering, the predominantly doublet NKT_Naive cluster was excluded, leaving NKT_CD160 and NKT_CD56 as two coherent populations. Donor-level proportion comparisons within the CD8 compartment did not detect any significant inter-group subset differences after correction (minimum adjusted *P* = 0.098), which included the NKT_CD56 subset (**Figures 4A–C**). Donor-level cytotoxicity and exhaustion scores differed across selected effector-associated subsets, with the effect direction and adjusted *P* values presented in revised **Figure 4D**. Changes in apoptosis and migration scores are reported as transcriptional signatures, and do not confirm cellular trafficking or activation-induced death *in vivo* (**Figure 4I**).

**Figure 4:**
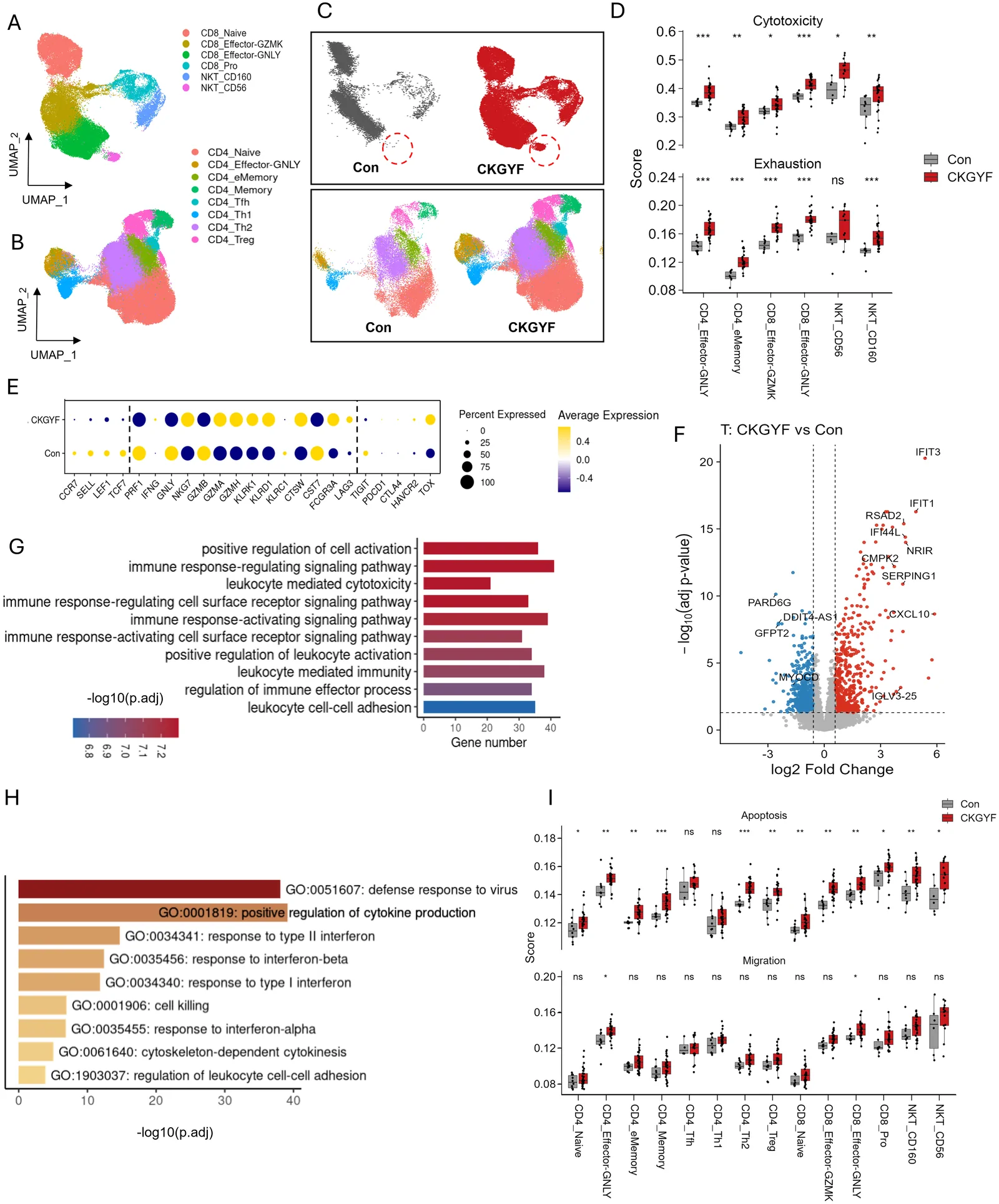
T-cell transcriptional states and functional gene-expression signatures in acute CHIKV infection. **(A, B)** UMAPs of the CD8-associated and CD4-associated compartments, respectively, coloured by annotated transcriptional clusters. Clusters labelled NKT_CD160 and NKT_CD56 retain the naming used in the figure. **(C)** Condition-separated embeddings of the two compartments; dashed circles mark the highlighted cluster in the upper embeddings. **(D)** Donor-level cytotoxicity and exhaustion module scores in selected effector-associated populations. **(E)** Expression of selected genes by condition; dot size denotes the percentage of expressing cells and colour denotes scaled average expression. **(F)** Differential expression in T cells, comparing CHIKV with controls; red and blue indicate increased and decreased expression, respectively. **(G, H)** Gene Ontology biological-process enrichment summaries, displaying gene counts **(G)** and -log10 (adjusted P) values (H); the colour scale in G also encodes enrichment significance. **(I)** Donor-level apoptosis and migration module scores across the indicated clusters. Module scores describe transcriptional programmes rather than direct measurements of cell killing, exhaustion, apoptosis or migration. The study cohort comprised 10 controls and 29 patients.

### 2.5 CHIKV infection enhances monocyte-centered immune cell communication networks

To investigate the potential intercellular relationships and transcriptomic alterations, the differences in cellular interactions among cells in CHIKV-infected and uninfected samples were analyzed using CellChat. Following infection, monocytes emerged as the central signaling hub, demonstrating significantly increased incoming and outgoing communication strength, as well as a higher frequency of communication events, particularly with B cells, plasma cells, NK cells, and γδ T cells (**Figures 5A, B**). In the CKGYF group, TNF signaling from monocytes to CD8^+^ T and B cells was notably enhanced, and the SIGLEC1-SPN ligand-receptor interaction from monocytes to other immune cells was also intensified, potentially facilitating immune cell recruitment and activation during infection (**Figure 5C**). Comparative analysis of monocyte-mediated signaling pathways revealed a significant upregulation of pathways related to inflammation, immune recruitment, and immune regulation (such as CXCL, BAFF, TNF, IL, ICAM/SELPLG), underscoring that CHIKV infection induces heightened monocyte-driven inflammatory and immunoregulatory signaling (**Figure 5D**). We conducted an in-depth analysis of the principal communication pathways originating from monocytes and discovered that pathways related to MHC-I and MHC-II were predominantly influenced by interactions between various HLA-CD8 and HLA-CD4 molecules. This finding suggests that antigen presentation-mediated T cell activation constitutes a predominant mechanism of cel-cell communication under pathological conditions (**Figure 5E**).

**Figure 5:**
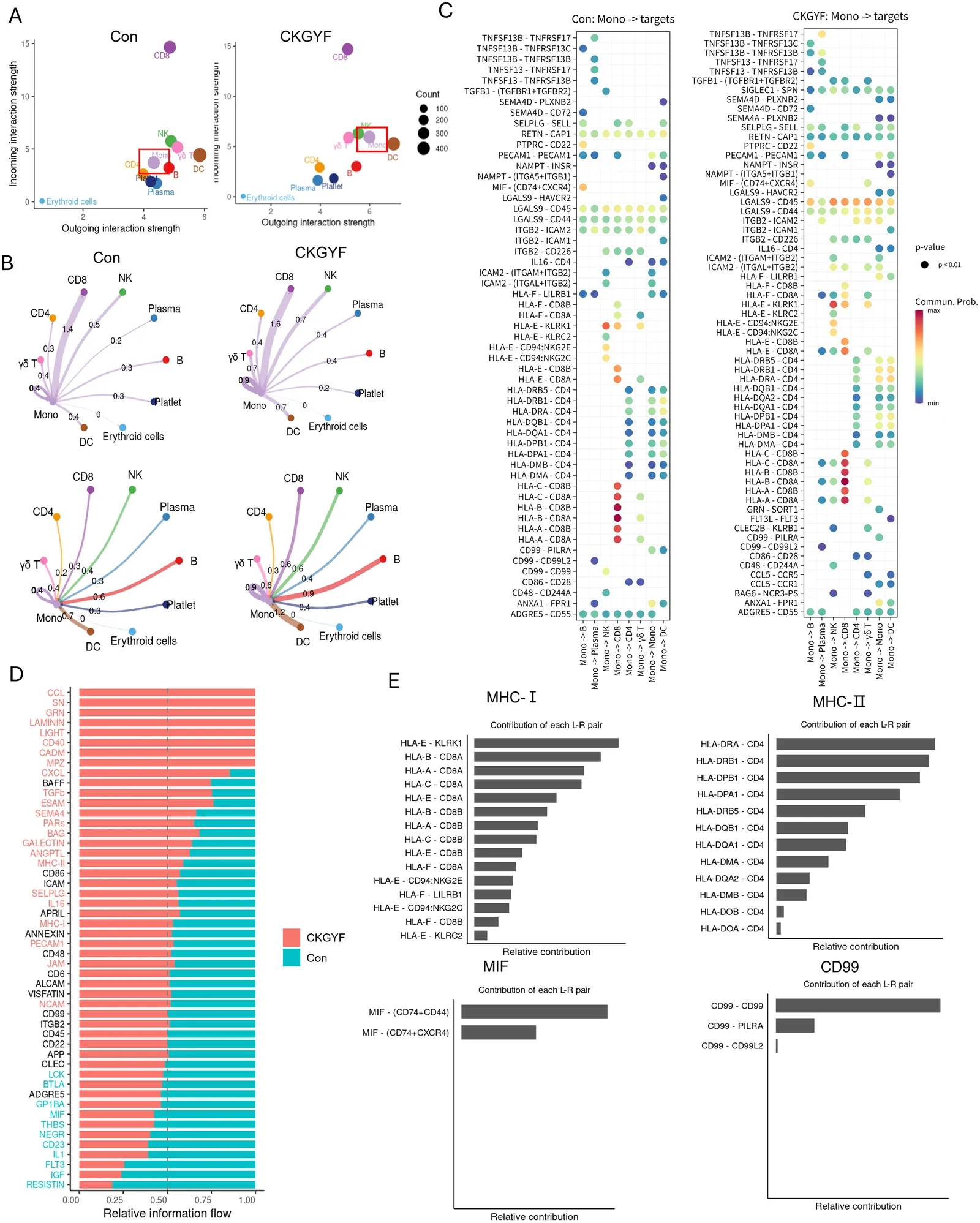
Inferred intercellular communication networks in acute CHIKV infection. **(A)** CellChat analysis of incoming and outgoing interaction strength for each population in controls (Con) and CHIKV-infected patients (CKGYF). Dot size represents the number of inferred interactions; boxes highlight monocytes. **(B)** Directed networks connecting monocytes with other populations. Arrows indicate inferred signalling direction, and numerical edge labels indicate interaction strength. **(C)** Ligand-receptor interactions with monocytes as senders and the indicated populations as receivers. Coloured dots denote interactions passing the displayed permutation threshold (P < 0.01); colour represents inferred communication probability. **(D)** Relative information flow for the indicated signalling pathways, partitioned between the two conditions. **(E)** Relative contributions of individual ligand-receptor pairs to the MHC-I, MHC-II, MIF and CD99 pathways. Communication networks were inferred from transcript abundance using CellChat.

### 2.6 Distinct antiviral immune responses and systemic validation of the interferon-driven state

After filtering cells with a minimum of 10 UMIs for total heavy chains, we evaluated immunoglobulin heavy-chain isotypes separately in B-cell subsets and plasma cells, and took non-B cell populations as the reference for ambient RNA. Among qualified cells, *IGHM* dominated in naive B cells, accounting for 97.0%, while plasma cells presented a mixed isotype profile (*IGHM* 28.0%, *IGHA1* 32.0%, *IGHG4* 23.2%; **Figure 6A**). We also analyzed seven publicly available DENV PBMC samples (GSE220969) together with the CHIKV cohort. After within-panel correction, donor-level ISG-score comparisons between the two datasets showed no statistical significance, and the contrast of monocyte inflammation score between CHIKV and DENV groups was also not significant (*P* = 0.142). Since we could not achieve clinical matching and uniform preprocessing across studies, the observed cross-study transcriptomic differences were interpreted descriptively, rather than as comparisons of relative response magnitudes (**Figures 6E–G**).

**Figure 6:**
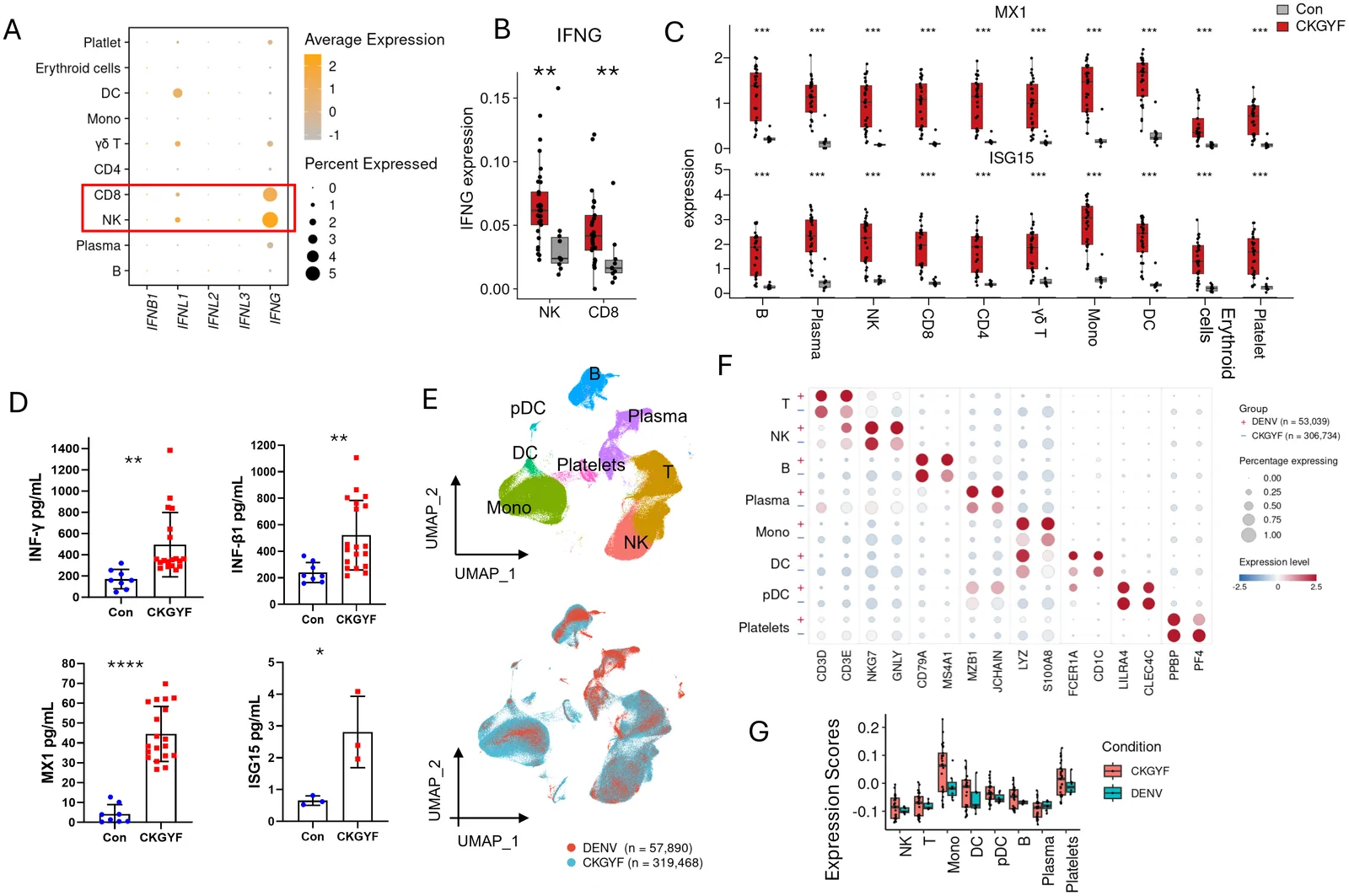
Interferon-associated transcription, plasma protein measurements and exploratory comparison with a DENV cohort. **(A)** Expression of IFNB1, IFNL1, IFNL2, IFNL3 and IFNG across PBMC populations; dot size indicates the percentage of expressing cells and colour indicates scaled average expression. The box highlights NK and CD8 T cells. **(B)** Donor-level mean IFNG expression in NK and CD8 T cells. **(C)** Donor-level mean MX1 and ISG15 expression across the indicated populations. **(D)** Plasma concentrations of IFN-gamma, IFN-beta1, MX1 and ISG15 measured by enzyme-linked immunosorbent assay. Assay samples and standards were measured in technical duplicate. **(E)** Cross-study UMAP coloured by cell identity (upper) and cohort (lower), combining CHIKV PBMCs with seven public dengue virus (DENV) samples from GSE220969. **(F)** Marker expression across populations and cohorts; dot size represents the fraction of expressing cells and colour represents scaled expression. **(G)** Donor-level interferon-stimulated-gene module scores in the CHIKV and DENV cohorts. Cross-study comparisons are exploratory because clinical characteristics and upstream processing were not matched.

To independently corroborate the robust interferon-stimulated transcriptomic signatures identified in silico at the systemic level, we evaluated both mRNA expression in bulk PBMCs and circulating protein concentrations in patient plasma. qPCR analysis demonstrated a significant upregulation in the mRNA levels of type I and II interferons (*IFNB1*, *IFNG*) and classical interferon-stimulated genes (*MX1*, *ISG15*) in PBMCs from patients infected with CHIKV compared to healthy controls. Remarkably, consistent with this transcriptional upsurge, our concurrent ELISA analysis confirmed that the circulating protein concentrations of IFN-β1, IFN-γ, MX1, and ISG15 were significantly elevated in the patient cohort (**Figure 6D**). Notably, the systemic increase in IFN-γ reflects the enhanced effector functions of CD8^+^ T and NK cells identified in our single-cell atlas, while the pronounced induction of IFN-β1, MX1, and ISG15 supports the widespread antiviral state primarily mediated by the monocyte network. Collectively, this orthogonal validation from transcript to protein provides compelling evidence that acute CHIKV infection triggers a substantial, systemic interferon-driven inflammatory response.

## 3 Discussion

In this study, we conducted a comprehensive characterization of the peripheral immune landscape associated with CHIKV infection at a single-cell resolution, utilizing PBMC samples from both healthy individuals and infected patients. Our findings demonstrate that CHIKV infection alters the relative composition of the circulating immune compartment. However, after multiple-testing correction, donor-level comparisons of PBMC population proportions did not retain statistical significance, meaning we cannot infer the absolute expansion or depletion of any immune population. Across major cell types, there was a widespread elevation of interferon-related signatures, suggesting that CHIKV infection triggers a systemic antiviral transcriptional program in peripheral blood (**Figures 4F, H**, **6B, C**). Notably, among innate immune cells, monocyte subsets exhibited the most robust inflammatory and interferon-stimulated responses, underscoring the pivotal role of monocytes in orchestrating the host’s antiviral defense during CHIKV infection. Within the B-cell lineage, CHIKV infection is associated with clear changes in plasmablast proportions and immunoglobulin heavy-chain profiles. These alterations are consistent with modified humoral states, but do not confirm the occurrence of lineage transitions or the generation of CHIKV-specific binding antibodies. Within the T-cell compartment, we observed heightened cytotoxicity and exhaustion signatures, notably within effector-associated T/NK-like T cell subsets (**Figures 4E, G**).

These observations collectively imply that CHIKV infection induces interferon-mediated activation of innate immune responses, thereby enhancing antiviral defense strategies. This response pattern parallels the innate immune activation observed in PBMCs during COVID-19 infection (14). Furthermore, DEGs in monocytes were particularly enriched in pathways associated with the positive regulation of cytokine production, suggesting that activated monocytes may further influence immune responses through cytokine modulation.

Simultaneously, there was a notable upregulation of IGHG1, IGHG3, and IGHG4, while IGHA1, IGHA2, and IGHG2 exhibited downregulation in the cohort infected with CHIKV. Notably, IgG1 and IgG3 are well-recognized for their potent antiviral effector functions and their capacity to activate complement pathways (15, 16), whereas IgG2 is primarily associated with responses to bacterial capsular polysaccharide antigens (17). Furthermore, IgG4 is characterized by its immunomodulatory properties (18). Taken together, these findings indicate that CHIKV infection is accompanied by altered immunoglobulin isotype profiles, but heavy-chain transcript isotype alone is insufficient to confirm antigen specificity or antiviral function.

The observed increase in plasma cell proportions, accompanied by a reduction in the overall B-cell population, is consistent with accelerated differentiation of B cells into antibody-secreting cells. However, due to the cross-sectional study design, we cannot confirm this directional transition. Such a phenomenon is frequently documented in arboviral infections, where rapid humoral responses are initiated during the acute phase. For instance, research on dengue virus infection has demonstrated that the infection can induce the expansion of CD14^+^, CD16^+^monocytes, which subsequently promote B-cell differentiation into plasmablasts and plasma cells (19). Furthermore, the enrichment of platelets and erythroid cells (presumably nucleated erythroid progenitors), along with enhanced antiviral and inflammatory responses, suggests that platelet activation (20) and stress hematopoiesis (21) may occur during CHIKV infection. Overall, our findings suggest that CHIKV infection triggers a coordinated immune response characterized by robust innate antiviral activation and dynamic remodeling of the adaptive immune system.

A notable discovery of this study is the identification of monocytes as the central communication hub within the CHIKV immune network. Utilizing CellChat (22) analysis, it was revealed that CHIKV infection substantially enhances monocyte-centered signaling, with significant amplification of the TNF, CXCL, BAFF, and IL pathways. These findings contribute to the current understanding of CHIKV immunopathogenesis by suggesting that monocytes serve not only as transcriptionally activated responders but also as crucial coordinators of immune communication in the peripheral circulation. Furthermore, our exploratory cross-study comparison with a public DENV dataset (11) revealed shared and dataset-specific patterns. Because the cohorts were not clinically or technically matched, this comparison was not used to rank response magnitude between the two viruses.

In addition, our orthogonal validation, which integrates cellular transcriptomics with circulating protein phenotypes, establishes a robust framework for elucidating systemic CHIKV pathology. The remarkable concordance between the upregulated monocyte-driven ISG network identified through scRNA-seq and the systemic elevations of MX1 and ISG15 quantified via qPCR and ELISA is particularly noteworthy. While traditional clinical assessments of CHIKV primarily focus on broad inflammatory markers (23), our findings suggest that circulating *MX1* and *ISG15* may serve as candidate, mechanistically grounded biomarkers for evaluating the intensity of the early innate antiviral response. Furthermore, given the elevated migration scores and enhanced chemotactic signaling, such as the CXCL network (24, 25), observed in these highly inflammatory monocytes, we propose that these peripheral cells function as a “vanguard” population. Upon systemic activation, these circulating inflammatory monocytes may efficiently migrate to local musculoskeletal sites, potentially contributing to the synovial inflammation and severe polyarthralgia that are clinical hallmarks of CHIKV infection (26, 27).

Healthy controls were selected on the basis of routine health examination and absence of a history of CHIKV infection or febrile symptoms. CHIKV serological screening was not available for asymptomatic controls; therefore, unrecognized prior or recent infection cannot be completely excluded. No convalescent, chronic, or longitudinal follow-up samples were available in the present cohort. Therefore, the persistence or resolution of the identified immune signatures after the acute phase cannot be determined from this study. Despite these insights, it is important to acknowledge several limitations inherent in this study. Firstly, the analysis was conducted using PBMC samples, which, while providing a convenient overview of systemic immune responses, do not fully capture the tissue-specific immune events occurring at local sites of viral replication and pathology, such as the joints. Secondly, the study employed a cross-sectional design, which restricts our ability to elucidate the temporal dynamics of immune responses throughout the course of infection and recovery. Thirdly, although multiple transcriptional alterations and inferred changes in cell-cell communication were identified, these developmental inferences remain fundamentally descriptive in the absence of pseudotime trajectory mapping or *in vitro* functional cellular assays. Lastly, the comparative integration with a public DENV dataset may be subject to analytical batch effects, despite the application of integrative methods. Additionally, the control group in this study was small, and paired absolute blood cell counts were not available, so the observed relative differences in cell composition cannot be interpreted as absolute expansion or depletion of any cell population. The comparison with DENV was conducted using seven selected samples without a documented selection protocol, and there was insufficient clinical or technical matching; furthermore, the CellChat interaction scores are still inferential rather than validated at the donor level. Therefore, these cross-study findings are exploratory only. Future research should adopt longitudinal multi-omics approaches and targeted functional studies to further refine pathogen-specific immune biomarkers.

## 4 Materials and methods

### 4.1 Ethical approval statement

This study was approved by the Institutional Ethics Committee of Foshan Women and Children’s Hospital (FSFY-MEC-2025-138), and written informed consent was obtained from all participants.Recruitment Period: August to September 2025. Participant-level demographic and clinical characteristics are summarized in **Supplementary Table S4**, including age, sex, comorbidities, days from symptom onset to sampling, and available virological and severity information.

### 4.2 Patient recruitment and sample preparation

This study received approval from the Institutional Ethics Committee of Foshan Women and Children’s Hospital (Approval No. FSFY-MEC-2025-138). Between August 2025 and September 2025, 29 confirmed cases of Chikungunya fever and 10 healthy control subjects were recruited. The inclusion criteria for patients infected with CHIKV were: (i) the presence of fever accompanied by severe arthralgia; (ii) a positive result for CHIKV RNA via real-time reverse transcription polymerase chain reaction (real-time RT-PCR) or a positive result for serum anti-CHIKV IgM antibody; and (iii) symptom onset within 7 days prior to enrollment. Healthy controls were individuals undergoing routine health examinations during the same timeframe, with no history of CHIKV infection or febrile symptoms. Peripheral venous blood samples (5 mL) were collected from each participant into EDTA-anticoagulated tubes. The samples were allowed to stand at room temperature for 30 minutes, followed by centrifugation at 3000 rpm for 10 minutes at 4 °C to separate the plasma from the cellular fraction. The plasma was aliquoted and stored at −80 °C for subsequent ELISA analysis, while the cellular fraction was utilized for the isolation of PBMCs.

### 4.3 scRNA-seq data processing, cell clustering, and dimension reduction

Single-cell RNA-seq libraries were prepared using 10x Genomics Chromium Single Cell 3’ (v3.1) with kit PN-1000121/DNBelab C4 (MGI).

Provider quality-control metrics across the 39 libraries were as follows: estimated number of cells 11,534 (range 2,284–18,171) per library; mean reads per cell 37,391 (range 24,487–214,744); mean UMI counts per cell 6,366 (range 4,789–9,602); sequencing saturation 80.5% (range 64.2–96.2%); fraction of reads in cells 84.6% (range 74.0–88.7%); and Q30 bases across cDNA reads 95.0% (range 92.1–96.2%). In total, 418,209 cells were recovered across the 39 libraries; after quality control filtering, 414,257 cells were retained for downstream analysis. Per-sample values are given in **Supplementary Table S1**. Ambient background was assessed with CellBender as documented in the original analysis; cells with 200–6,000 detected genes and <15% mitochondrial reads were retained.

Doublets were detected independently within each sample using scDblFinder with default parameters. Cells classified as doublets were excluded from the whole atlas and downstream subsets using the same barcode-level calls. Approximately 11% of atlas cells were removed. The NKT_Naive cluster was additionally excluded from T-cell analyses because approximately 94% of its cells were classified as doublets; NKT_CD160 and NKT_CD56 were retained after post-filtering reclustering.

The 39 samples were integrated for visualization and clustering in Seurat v5 using CCA, 2,000 highly variable genes and 30 principal components. Cluster identities were assigned by manual inspection of canonical marker expression (for example, *CD3D*/*CD3E* for T cells, *NKG7*/*KLRF1* for NK cells, *CD79A*/*MS4A1* for B cells and *CD14*/*LYZ* for monocytes), followed by cluster renaming.

### 4.4 Analysis of differentially expressed genes

Cluster markers were identified using MAST. For between-condition differential expression analysis, raw UMI counts were summed across all cells from each donor within each cell population, and then analyzed with DESeq2 under the design ~ group. Donors contributing fewer than ten cells to a given population were excluded from that specific comparison. Genes were retained only if they had at least ten counts in at least two donor-level pseudobulk samples. Differentially expressed genes were defined as those with a Benjamini–Hochberg-adjusted *P* < 0.05 and |log2 fold change| ≥ 0.58, and the biological replicate in this analysis was the donor rather than the individual cell.

For each module-score or expression comparison, cell-level values were averaged within donor and compared between groups by a two-sided Wilcoxon rank-sum test. Rank-biserial correlation and the between-group mean difference were reported; 95% confidence intervals for mean differences were obtained from 2,000 donor-level bootstrap resamples (seed 1234). For revised cell-composition panels, each donor’s cell-type proportion was logit-transformed with an offset of 10−4 and compared using Welch’s t-test; percentage-point differences and bootstrap 95% confidence intervals were reported. *P* values were adjusted by the Benjamini–Hochberg method within each panel. Pooled observed-to-expected ratios were descriptive only.

Module scores were calculated using Seurat::AddModuleScore on the log-normalized RNA assay data layer, with the parameters set as nbin = 24, ctrl = 100, k = FALSE, and seed = 1. For each gene set, we retained only genes that were detected in the analysis object; full gene lists, gene set sources, overlap information, and the number of detected genes are provided in **Supplementary Table S2**. For B-cell inflammatory scoring, *S100A8*, *S100A9*, *S100A12*, *CD14*, *FCN1*, *LYZ*, *VCAN*, *HBB*, *HBA1*, and *HBA2* were excluded *a priori*. Immunoglobulin isotype was assigned based on the most abundant heavy-chain constant-region transcript, and this assignment was only performed for cells with a total heavy-chain UMI count of at least ten. The same rule was applied to non-B cells to generate an ambient reference. Isotype distributions were included for descriptive purposes only and were not interpreted as indicators of antigen specificity.

Seven DENV PBMC samples from GSE220969 (GSM6833291, GSM6833293, GSM6833295, GSM6833296, GSM6833302, GSM6833303 and GSM6833304; **Supplementary Table S3**) were included in an exploratory cross-study analysis. The available script applied log normalization, selected 2,000 variable genes, used 30 principal components and ran Harmony by sample identity. ISG scores were calculated from the log-normalized RNA data layer. The selection of these seven samples from the public series was not documented, and age, illness day and severity were unavailable in the files used; the DENV data were not processed with the same upstream ambient-RNA and quality filters as the CHIKV data. Consequently, donor-level contrasts were interpreted descriptively and did not establish virus-specific response magnitude.

### 4.5 Gene ontology enrichment analysis

To elucidate the biological functions of the identified DEGs, a GO term enrichment analysis was conducted using the clusterProfiler R package (28). The functional annotations and subsequent visualizations were specifically confined to the GO Biological Process (GOBP) categories.

### 4.6 Cell-cell communication analysis

To deduce and examine intercellular communication networks, ligand-receptor interaction analysis was conducted utilizing the CellChat R packag (29). Overexpressed ligands and receptors identified within distinct cell populations were mapped onto a protein-protein interaction (PPI) network. CellChat extrapolates biologically significant cell-cell communications by assigning probability values to each interaction and conducting rigorous permutation tests. The resultant cellular communication networks and specific signaling pathways were visualized employing conventional circle and bubble plots.

### 4.7 RNA extraction and quantitative real-time PCR

To validate the transcriptomic findings, total RNA was extracted from patient and control PBMCs using TRIzol reagent according to the manufacturer’s protocol. The RNA was reverse-transcribed into cDNA. qPCR was performed using SYBR Green master mix on an ABI 7500 Real-Time PCR system. The relative mRNA expression levels of target genes (IFNG, IFNB1, MX1, and ISG15) were calculated using the 2-ΔΔCt method, with GAPDH or ACTB serving as the internal reference gene. Due to available RNA yield limits, qPCR validation was performed on a representative subset of the original cohort.

### 4.8 Enzyme-linked immunosorbent assay validation

ELISA was utilized to quantify the concentrations of IFN-γ, IFN-β1, MX1, and ISG15 proteins in plasma samples. The frozen plasma samples underwent centrifugation, and the assays were conducted in accordance with the manufacturers’ standard protocols. Absorbance readings were taken at 450 nm using a microplate reader. Standard curves were constructed employing a four-parameter logistic regression model to determine the concentrations of the target proteins, with all samples and standards analyzed in duplicate. Owing to the limited plasma volumes available from the acute cohort, ELISA measurements for certain targets, such as ISG15, were performed on representative subsets of the study population, as indicated in the corresponding figure legends.

### 4.9 Statistics and code availability

All statistical analyses and visualizations were performed using R. Between-group comparisons of module scores and gene expression were conducted using two-sided Wilcoxon rank-sum tests; comparisons of cell composition were carried out using logit-transformed donor proportions and Welch’s t-tests; pseudobulk differential expression analysis was implemented with DESeq2, as described earlier. Significance is denoted by the following symbols: ns: *P* > 0.05; *: *P* ≤ 0.05; **: *P* ≤ 0.01; ***: *P* ≤ 0.001; ****: *P* ≤ 0.0001.

## Data Availability

The datasets presented in this study can be found in online repositories. The names of the repository/repositories and accession number(s) can be found in the article/**Supplementary Material**. All analysis code is available at: https://github.com/wlzmd/CHIKV-scRNAseq. Experimental data have been uploaded to the GSA (Genome Sequence Archive) database (URL: Genome Sequence Archive- CNCB-NGDC dataset; accession number: HRA017257).
